# When is enough, enough? Quantifying trade-offs between information quality and sampling effort for fishing gear selectivity data

**DOI:** 10.1371/journal.pone.0199655

**Published:** 2018-06-21

**Authors:** Tiago Veiga-Malta, Jordan Feekings, Bent Herrmann, Ludvig Ahm Krag

**Affiliations:** 1 DTU Aqua, Technical University of Denmark, Hirtshals, Denmark; 2 SINTEF Fisheries and Aquaculture, Hirtshals, Denmark; 3 University of Tromsø, Breivika, Tromsø, Norway; Department of Agriculture and Water Resources, AUSTRALIA

## Abstract

There is general pressure throughout the world’s fisheries for the industry to have greater involvement not only in the development of fishing gears but also in the testing and documentation of their effect. In the European Union, the Common Fisheries Policy of 2013, together with the proposed reform of the technical measures regulation, highlights the need for greater flexibility in fisheries through increased stakeholder involvement. To achieve this flexibility, there is a need for additional fishing gears available to the fishermen. A way to facilitate this is to have the industry take part in the development and testing of fishing gears, as well as collect data on their performance. However, to have a successful industry-collected data programme, fishermen have to be able to collect data on the length of a portion of the catch. In this study, we determine how many individuals need to be measured to correctly evaluate the relative selective performance of a new gear compared to a standard gear. The evaluation was carried out by analysing catch ratio curves, their associated uncertainties, and the trade-offs between uncertainties and sampling effort. Results show that with relatively small sample sizes (500 to 1000 individuals) it is possible to correctly evaluate the performance of a gear for a given species. By having the industry develop and test their own gears, as well as being involved in the collection of data, the number of potential gear solutions available to address the different issues emerging in the fisheries is increased.

## Introduction

Throughout the world’s fisheries there is a general trend for the industry to have a greater involvement, not only in the development of fishing gears, but also in the testing and documentation of their effect [1]. In the European Union, the Common Fisheries Policy (CFP) of 2013, where the catch of all listed species are required to be landed and counted against quota [2], introduces additional pressure on the industry to eliminate or considerably reduce unwanted catch [3]. This unwanted catch can either be species- or size-specific, and its composition can vary depending on the quotas available to each fishing vessel. Thus, the ability of fishermen to adjust the selectivity of their gear to suit the quotas which are available to them will be an important factor in determining the revenue and profitability of their fishery. As the combination of gear, fishing practice and quota shares will differ between fisheries and vessels, changes to the selectivity of the gear will need to be applied quickly and at a vessel level. However, to achieve such flexibility a greater number of more specific gear solutions is needed.

Under current EU management, such flexibility is limited, where only a couple of generic gears are legislated for each gear group and area. The current setting is often slow and inflexible, where few fishing gear solutions are tested and new gears take several years to be implemented in legislation. Moreover, the “one gear fits all” type of legislation further reduces the system’s flexibility and capacity to adjust to upcoming problems. This lack of flexibility in the legislative framework has been highlighted in the proposed reform of the technical measures regulation as one of the major shortcomings of the current regulation [4].

To achieve the necessary flexibility, there needs to be a framework where a greater number of new gears or modifications to existing gear can be adequately developed, tested, and their selectivity correctly documented. One way to facilitate this flexibility, and to reduce the economic and time outlay associated with the development of fishing gears, is to have the industry not only identify the problems but to develop and test the technical solutions themselves. Increasing the involvement of stakeholders in developing specific conservation measures (e.g. the development of gears) is also something which the proposed reform of the technical measures regulation aims to achieve [4]. The involvement of fishermen in gear selectivity projects, where they are an integral part of the process, has previously been shown to provide valuable experience-based knowledge [5–7]. Furthermore, having the industry identify the problems and test potential solutions helps incorporate them into the process, while also shifting the burden of proof onto the industry. Additionally, the involvement of fishermen in the development of fishing gears allows for a period where promising solutions can be identified and tested in a commercial setting before carrying out a rigorous scientific test. This also allows for the possibility of testing numerous fishing gears in parallel, as well as establishing a real commercial development and testing phase prior to expensive scientific trials. Furthermore, having the industry assist with the collection of data is a cost-effective solution since it avoids the need for scientific staff on board during development periods [8,9].

Despite there being several studies which have shown the validity of industry collected data [8–12], some concerns remain about the possible bias of such data [11–15]. Potential bias can occur if fishermen do not understand why the data are collected, sampling training is lacking and/ or the workload associated with sampling is excessive [9,11,12,16]. While the first two concerns can be addressed through good communication, the issue of excessive sampling effort is not so straightforward. Thus, to have a successful industry-collected data programme, those involved need to be burdened as little as possible. This is because they have another objective, which is to carry out a viable fishery. Therefore, in terms of industry-collected data, a relatively limited sample needs to be able to correctly quantify the performance of a new gear.

In this study, we address the issue of excessive sampling effort and how to minimize this burden by defining how many individuals need to be measured to correctly evaluate the selectivity of a new fishing gear relative to an existing gear. Since the selectivity of a gear is typically different for different sizes of a species, the relative selectivity between the two fishing gears needs to explicitly account for fish size. The relative selectivity can be expressed using the catch comparison method [17] that quantifies the length-dependent relative selectivity between two fishing gears. This method is particularly suitable to industry-collected data as it does not interfere with commercial fishing practices since the two gears are fished in parallel in a twin-rig setting. Previous studies have looked into the issue of how many individuals need to be measured to evaluate the selective performance of a new gear design for covered codend and paired-gear methods [18, 19]. However, such methods are not appropriate for industry-collected data, since they can interfere with the fishing activities. Therefore, it is relevant to address this issue using the catch comparison method. Furthermore, by understanding the trade-offs between sampling effort and gain in information, one can define the number of individuals needed to achieve a given accuracy, and thus avoid excessive data collection.

## Material and methods

**Fig 1:**
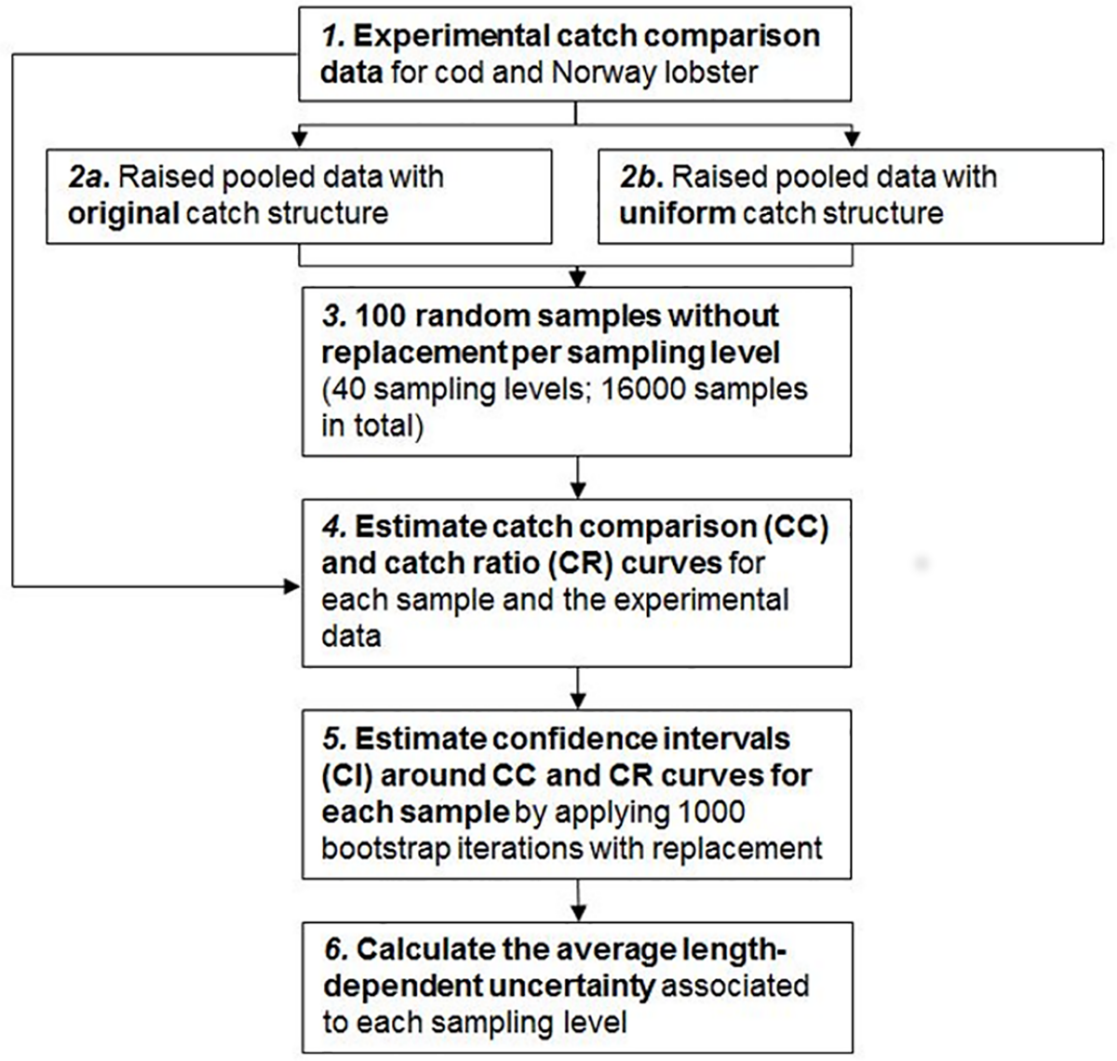
Flowchart depicting the main steps of the simulation procedure used in the present study.

In the present study, we investigate how many fish need to be length measured to correctly assess the catch performance of one gear in relation to another in a twin-trawl setting. A theoretical approach was chosen to investigate this topic, where catch comparison data were simulated based on experimental data. To facilitate the description of the different steps used in this simulation study a flowchart is presented in Fig 1. Since the study is solely based on simulated data there were no ethical concerns and thus no permits were required.

### Estimating catch comparison and catch ratio curves

To compare the performance of a new trawl to an existing one, the number of individuals of each length class caught in each codend was used to evaluate the length-dependent relative catching efficiency of both gears (step 4 in Fig 1). This was done by calculating the catch comparison rate, CC_*l*_, using the following equation:

$${CC}_{l}=\frac{{nt1}_{l}}{{nt1}_{l}+{nt2}_{l}}$$ (1)

where *n*t1_*l*_ is the number of fish of length *l* of the given species collected in the codend of the test gear and *n*t2_*l*_ is the number collected in codend of the standard (baseline) gear. CC_*l*_ ranges from 0.0 to 1.0, where a value of 0.5 implies that both gears have equal catch efficiency for a specific length, on the condition that both gears are fishing equally. One drawback of the catch comparison rate is that it is not possible to immediately infer the relative catch efficiency of the new gear compared to the standard gear. Alternatively, catch ratio, CR_*l*_, provides such information by comparing directly the catch efficiency at length for both gears [20]:

$${CR}_{l}=\frac{{nt1}_{l}}{{nt2}_{l}}$$ (2)

where a CR_*l*_ value of 1 means the catch efficiency for both gears, at length *l*, are equal. Thus, a CR_*l*_ = 1.25 would mean that the test gear caught on average 25% more at length *l* than the standard gear. In contrast, a CR_*l*_ = 0.75 would mean the test gear caught 25% less at length *l* than the standard gear. CR_*l*_ ranges from 0 and any positive value. Therefore, in this study, catch ratio was chosen as the relative catch efficiency descriptor.

In catch comparison analyses, CC_*l*_ is often modelled following the formula [21]:

$$CC(l,v)=\frac{exp(f\;(l,v_{0},\ldots ,v_{k}))}{1+exp(f\;(l,v_{0},\ldots {,v}_{k}))}$$ (3)

where *f* is a polynomial of order *k* with coefficients *v*_0_ to *v*_*k*_ where *v* = (*v*_0_,…,*v*_*k*_). *f* was considered up to an order of 4 with parameters *v*_0_, *v*_1_, *v*_2_, *v*_3_ and *v*_4_. Leaving out one or more of the parameters *v*_1_,…,*v*_4_ led to an additional 31 models considered as potential models for the catch comparison function *CC*(*l*,*v*). The final model selection was determined through a multimodel inference approach [22]. In this approach, rather than choosing the “best model” fit based on the Akaike's Information Criterion (AIC) value [23], the models are weighted by their respective AIC values. Here, all models where the difference between the respective AIC values and the lowest AIC value are no larger than 10, are used [24]. This method allows for an overall best estimation of the parameters of the model and their associated uncertainties. To estimate the catch ratio curves, Eq (1), Eq (2) and Eq (3) were combined in order to obtain the catch ratio curves directly from the catch comparison curves [20]:

$$CR(l,v)=\frac{CC(l,v)}{1-CC(l,v)}.$$ (4)

The Efron percentile 95% confidence intervals (CI) for the catch comparison and catch ratio curves were estimated using the bootstrapping method described by Efron [25]. A total of 1000 single bootstrap iterations with replacement were performed on each sample for each sampling effort level (described below) to estimate the 95% confidence intervals around the respective catch comparison and catch ratio curves (step 5 in Fig 1). A significant difference between the selective performance of the test and baseline gears occurs when either the upper or the lower limit is under or above the values for equal performance of the gears. These values are 0.5 and 1.0 for the CC and CR curves, respectively. The catch comparison and catch ratio analyses described above were performed using the statistical analysis software SELNET [26].

### Estimating uncertainties associated to each sampling effort level

In this study, relative uncertainties (henceforth, uncertainties) were used to directly compare the uncertainty associated to the CR(*l*,*v*) obtained from the different sampling levels (step 6 in Fig 1). These uncertainties were calculated for each length class by using the ratio between the 95% CI range and the catch ratio value obtained from the original data (described below). The average uncertainty for each length class and sampling level was used to define the expected uncertainty for each length class and sampling level. Subsequently, the relationships between the uncertainties within a specific length class and sampling effort were modelled using power models, since this was the expected behaviour for this relationship [18], using the following equation:

$$\hat{U}=a\times n^{b}$$ (5)

where $\hat{U}$ is the uncertainty for each length class, *a* and *b* the parameters for the respective power models and *n* the sampling level. Since the objective of this study was to understand the relationship between uncertainties and the total number of individuals sampled, and not the relationship between uncertainties and the number of individuals sampled in a specific length class, the total number of individuals per sample *n* was used. Regarding the fitting process of the power models, the model error distribution (additive vs. multiplicative) determines which model fitting method is more appropriate (linear regression on log-transformed data vs. non-linear regression on raw data; [27]). Therefore, we evaluated the error distribution following the approach suggested by Xiao *et al*. [27]. The analysis showed that the assumption of multiplicative log-normal error was better supported (results not shown), thus the power models were estimated by fitting a linear regression to the log transformed values. The coefficient of determination (*R*^*2*^) was used to evaluate the quality of the power model fits. This value was directly obtained from the linear model function within the statistical package “*stats*” implemented in the R software [28].

### Determining the minimum sampling effort needed

Considering that the aim of this study is to propose a sampling effort which can facilitate the involvement of the industry in the gear development and testing stages, a range of values is defined. The range defined aims to ensure that the relative performance of the new gear would be, in most cases, correctly evaluated while ensuring that excessive sampling is avoided. Firstly, the lower bound of the sampling range was based on the cases where the small sample sizes conform with the results observed in the full dataset, i.e. when a significant effect in the full dataset was also observed in the smaller sample size. Secondly, the upper bound of the sampling range was defined based on the trade-off between sampling effort and the decrease in uncertainties around the catch comparison curves. For example, when an increase in sampling effort (measuring 100 more individuals) does not lead to a considerable improvement (>5%) in uncertainty.

### Simulated data from sea trials

In order to achieve the objective of this study, and due to the large amount of data required, it was necessary to simulate random samples of catch comparison data with different total numbers of sampled individuals (sampling effort levels). The original dataset was raised by a factor of 50 (step 2a in Fig 1). This step allowed us to simulate the random subsamples without replacement for the sampling levels larger than the original datasets, while maintaining the original catch structure. A second set of catch comparison data was simulated assuming a uniform catch structure, where all considered length classes had a total of 10000 individuals (step 2 in Fig 1). By simulating a uniform catch structure, the effect of the catch structure can be better understood. Furthermore, using the same catch structure for all considered species removed its effect from the analysis, and thus allowed for other potential factors to be better understood. Similar to the raising of the originals datasets, the number of individuals per length class was chosen to allow the simulation of random samples without replacement for the larger sampling levels. The split between individuals caught in the standard and test gears in the uniform catch structure datasets was calculated based on the CC_*l*_ values per length class in the original dataset and by applying Eq (1). The same sampling levels were chosen for both simulated catch distributions, and ranged from a total of 100 to 10000 sampled individuals, with increments of 100 individuals from 100 to 2500 individuals; increments of 250 from 2500 and 5000 individuals; and increments of 1000 from 5000 to 10000 individuals (step 3 in Fig 1). For each sampling level and catch structure, 100 random samples were simulated with an equal number of sampled individuals per gear [29], resulting in a total of 16000 simulated samples. Moreover, subsampling factors were obtained by calculating the ratio between total number of individuals of the subsample per gear and the total number of sampled individuals in the simulated datasets. Each of the 100 random samples per sampling level and catch structure represents the pooled data of a single catch comparison trial with a given total number of sampled individuals. The data simulations described above were carried out using R [28].

### Sea trial data

To ensure that the simulated samples were as realistic as possible, the random subsamples were simulated based on actual catch comparison data (step 1 in Fig 1). The data used for this study were initially reported by Krag *et al*. [17] and collected as part of a scientific gear selectivity trial in the North Sea. In this sea trial, the test gear had two common trawl modifications: a topless trawl and a square mesh escape panel. A twin-rig trawl setup was used, where the modified gear was towed in parallel to the standard gear, and the total number of individuals caught per length class was collected for both gears.

The present study included data for cod (*Gadus morhua*) and Norway lobster (*Nephrops norvegicus*), since both are important commercial species and a change in the selective performance of the new gear was expected for one species (cod) but not the other (Norway lobster) [17]. Moreover, for an easier visualisation and interpretation of the results, 3 representative length classes were chosen for both species: one where the number of individuals was abundant, one where the number of individuals was few, and one with intermediate values (40, 65 and 90 cm for cod and 35, 45 and 65 mm carapace length for Norway lobster).

## Results

The catch comparison and catch ratio analyses of the simulated data (16000 samples) took an average of 12 central processing unit (CPU) hours per sample, adding up to approximately 192000 CPU hours. The average speed of the CPUs used for this analysis was around 2.0 GHz.

**Fig 2:**
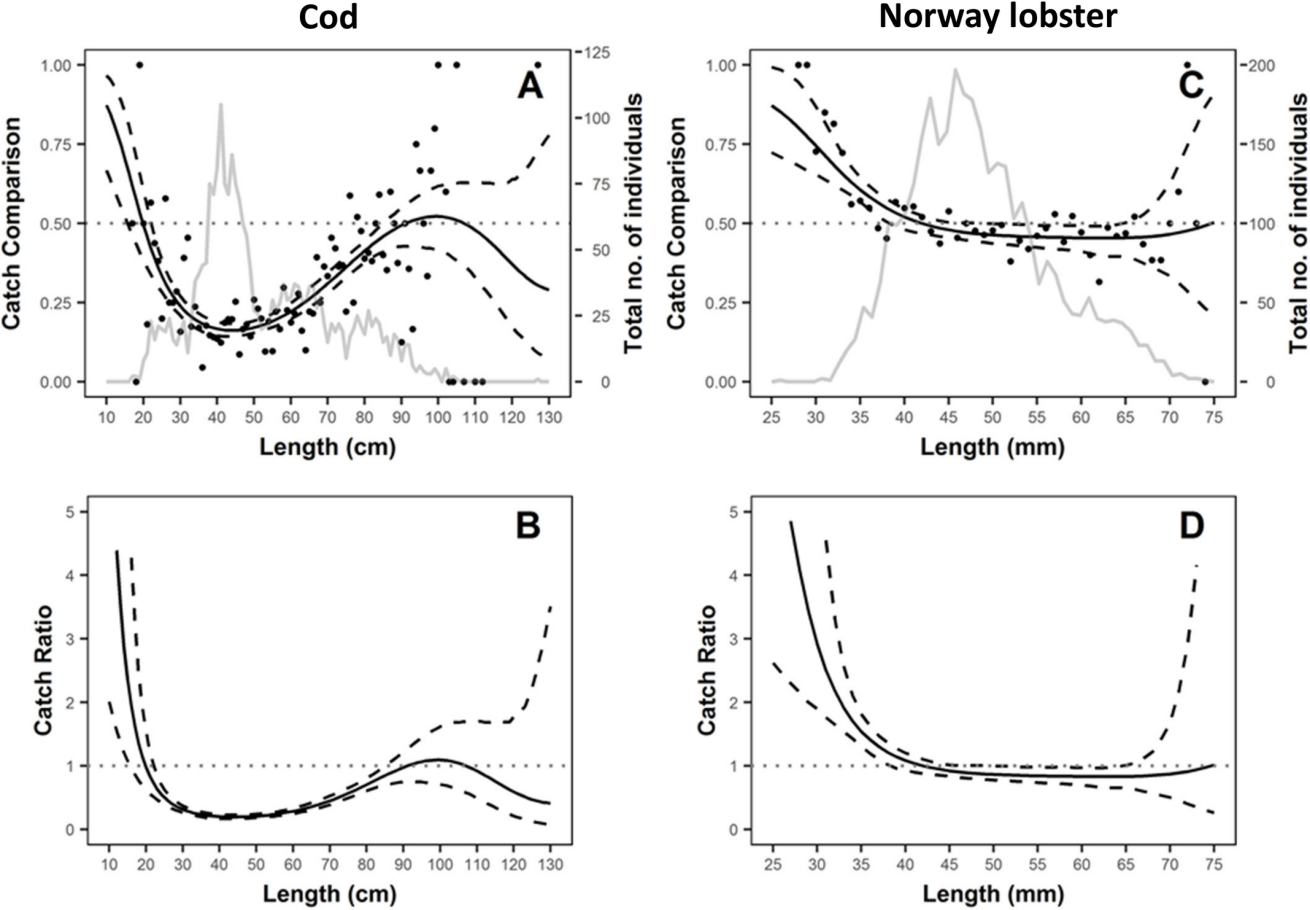
**Estimated catch comparison and catch ratio curves (solid black line) and 95% confidence intervals (broken black lines) for cod (A and B, respectively), and for Norway lobster (C and D, respectively)**. Dotted grey lines represent when both gears are fishing equally efficient. Grey solid lines in A and C represents the catch length structure for cod and Norway lobster, respectively.

The catch comparison and catch ratio results from the original data for cod and Norway lobster are shown in Fig 2. A significant length-dependent effect for both species was observed. The test gear caught significantly less cod between 23 and 84 cm, since for those length classes the catch comparison and catch ratio were significantly lower than 0.5 and 1.0, respectively (Fig 2A and 2B). Furthermore, a significant increase was observed for cod equal to or smaller than 15 cm. For Norway lobster, a significant increase was detected for individuals (< = 38 mm), while no significant effect was found for individuals larger than 38 cm (Fig 2C and 2D).

**Fig 3:**
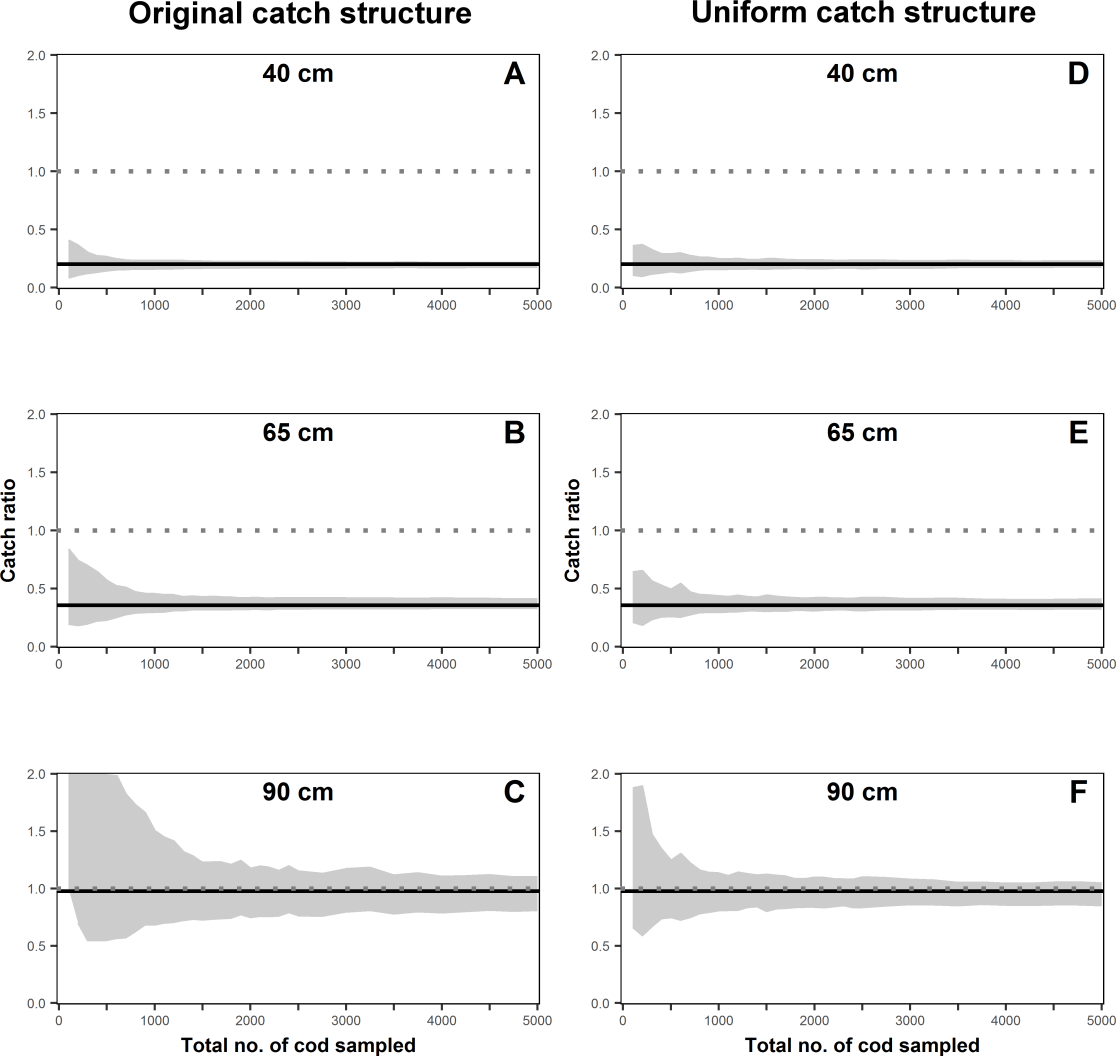
Effect of sample size on catch ratio confidence intervals (grey band) per length class for cod (40, 65, 90 cm). The confidence intervals shown in fig A to C are for the observed catch structure and from D to F are for the uniform catch structure scenario. The solid black line and the dotted grey line define for a specific length class the original catch ratio and when both gears are fishing equally efficient (catch ratio equal to 1), respectively.

The catch ratio values for cod from the simulated data were significantly different from 1.0 for the length classes of 40 and 65 cm in the observed catch structure (Fig 3A and 3B). The significant difference was observed even for the smallest sample size of only 100 individuals (Fig 3A and 3B). However, no significant effect was observed in the 90 cm length class, irrespective of the number of individuals measured (Fig 3C). For the uniform catch structure, the three chosen length classes for cod showed similar results. Furthermore, the CIs show a similar behaviour across all length classes, where the total range became narrower at smaller sample sizes than in the observed catch structure (Fig 3D, 3E and 3F).

**Fig 4:**
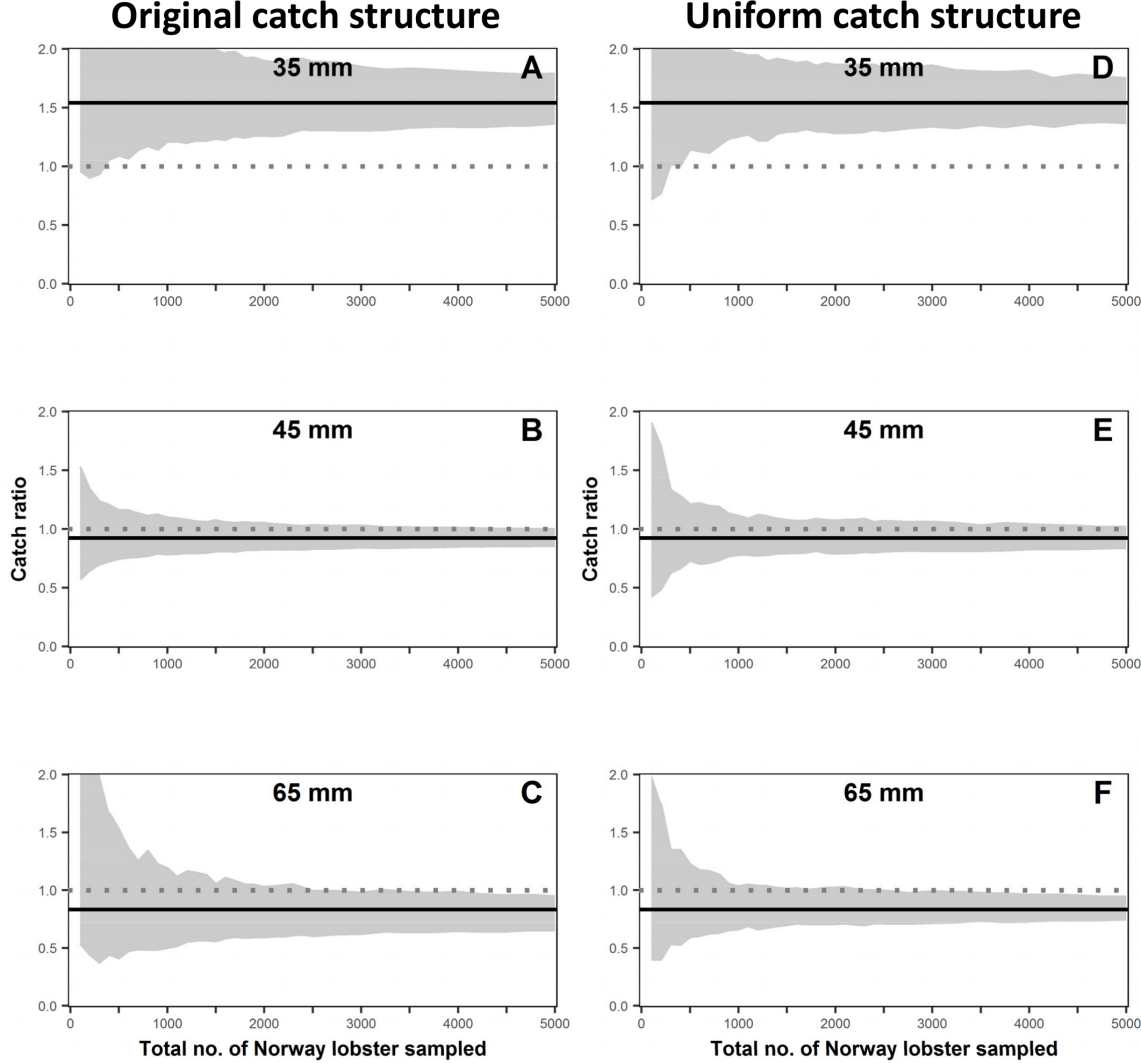
Effect of sample size on catch ratio confidence intervals (grey band) per length class for Norway lobster (35, 45, 65 mm). The confidence intervals shown in fig A to C are for the observed catch structure and from D to F are for the uniform catch structure scenario. The solid black line and the dotted grey line define for a specific length class the original catch ratio and when both gears are fishing equally efficient (catch ratio equal to 1), respectively.

For Norway lobster, catch ratios were significantly different from 1.0 for the 35 and 65 mm length classes (Fig 4A and 4C), while no significant difference was observed for the 45 mm length class (Fig 4B). For the 35 mm length class, a significant difference was observed at smaller sample sizes (a total sample size of ~350 individuals), while for the 65 mm length class a significant difference was observed only for larger sample sizes (a total sample size of ~3250 individuals). The CIs observed for Norway lobster with a uniform catch structure showed a similar behaviour to those observed for the original catch structure (Fig 4D, 4E and 4F). However, the total range became narrower at smaller sample sizes when compared to the CI ranges of the observed catch structure. This is similar to what was observed for cod.

In Table 1, the different parameters and the coefficients of determination (*R*^*2*^) for the different power models are shown. In general, the different fitted curves showed high *R*^*2*^ values, ranging from 0.88 to 1, with the lowest values observed in the tail areas of the chosen length classes for both species. This was more evident for the original catch structure. The curve describing the uncertainty for 90 cm cod from the original catch structure showed a tendency to predict with a systematic bias to higher values. However, this curve is also the curve with the lowest *R*^*2*^, thus indicating that the coefficient of determination was able to sufficiently describe the quality of the fits of the power model curves to the uncertainty data. Furthermore, there is a clear harmonisation of the quality of the fits for both species in the case of the uniform structure. A similar tendency was also observed for both parameters of the power models (*a* and *b*). For both species, the highest values (thus, higher uncertainties) were observed in the tail areas, but when the uniform catch structure was applied this difference was substantially reduced.

**Table 1:** Power model parameters (*a* and *b* from equation 6) for the relative uncertainties and goodness of fit per sampling level, length class, species, and catch length structure. Coefficient of determination (*R*^*2*^) represents the quality of the fit and ranges from 0 to 1, where 1 is a perfect fit.

		Original catch structure			Uniform catch structure		
Species	Length class*	*a*	*b*	*R*^*2*^	*a*	*b*	*R*^*2*^
**cod**	25	185.36	-0.76	0.90	18.09	-0.52	0.96
	30	90.15	-0.73	0.88	14.92	-0.49	0.95
	35	23.62	-0.58	0.97	13.93	-0.48	0.96
	40	16.04	-0.54	0.97	12.44	-0.46	0.96
	45	14.49	-0.53	0.96	10.84	-0.45	0.96
	50	19.37	-0.57	0.96	9.75	-0.45	0.96
	55	26.53	-0.60	0.95	9.61	-0.45	0.95
	60	29.47	-0.60	0.94	10.18	-0.46	0.94
	65	27.82	-0.59	0.93	11.02	-0.46	0.93
	70	27.13	-0.58	0.92	11.61	-0.47	0.92
	75	39.84	-0.63	0.93	11.67	-0.47	0.92
	80	54.46	-0.66	0.92	11.20	-0.48	0.92
	85	124.26	-0.74	0.92	10.58	-0.48	0.92
	90	200.19	-0.78	0.88	10.70	-0.48	0.93
**Norway lobster**	30	1381.48	-0.82	0.94	34.72	-0.58	0.94
	35	39.19	-0.59	0.97	22.71	-0.53	0.95
	40	11.37	-0.49	1.00	14.05	-0.49	0.96
	45	9.83	-0.49	1.00	12.79	-0.49	0.96
	50	10.72	-0.47	1.00	14.38	-0.50	0.96
	55	14.95	-0.50	0.99	13.92	-0.50	0.96
	60	25.72	-0.54	0.98	14.84	-0.51	0.96
	65	38.69	-0.55	0.98	19.07	-0.52	0.96
	70	12627.31	-1.13	0.89	34.63	-0.59	0.89

*Please note that length classes for cod are in cm (total length) and for Norway lobster in mm (carapace length)

**Fig 5:**
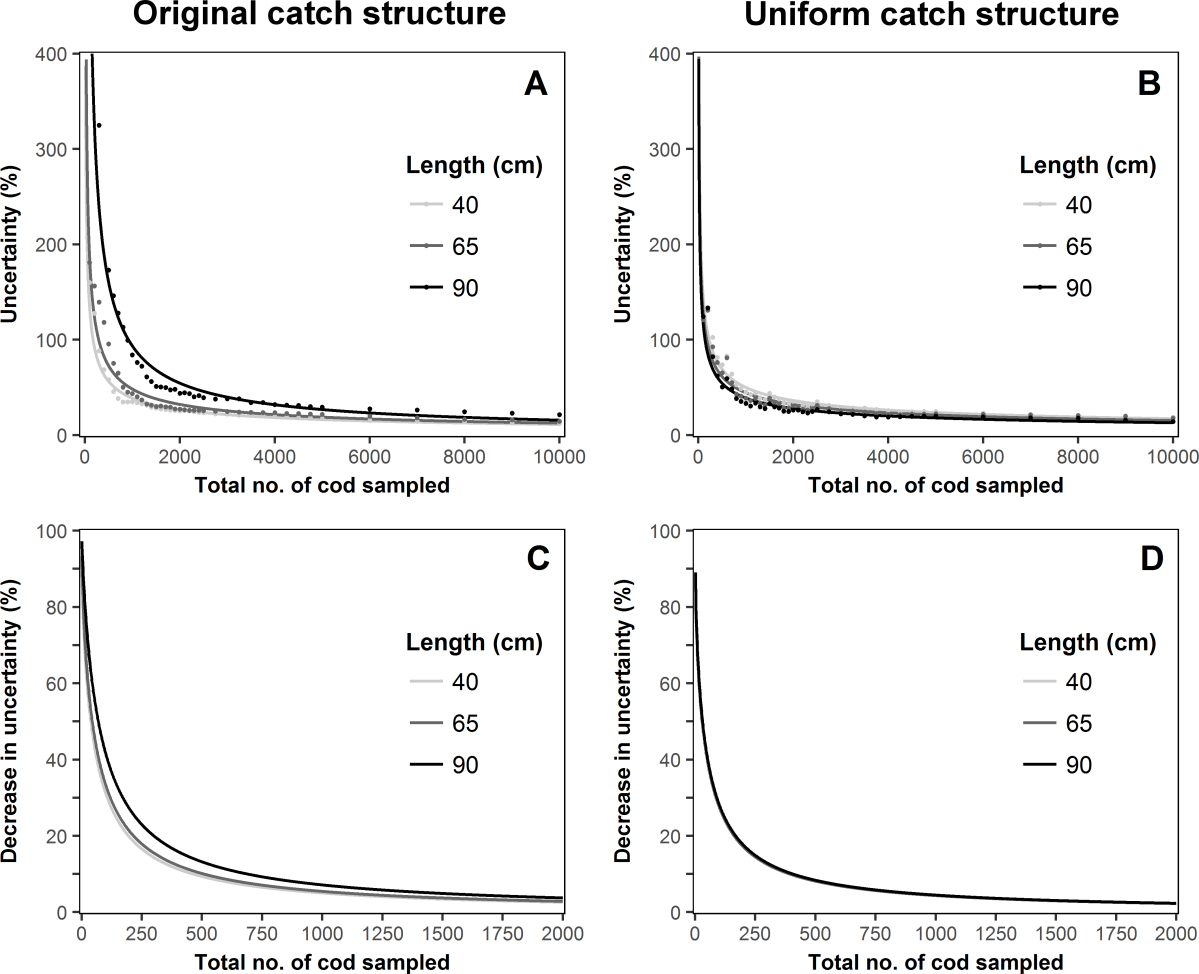
**Cod uncertainty curves for the observed catch structure (A) and uniform catch structure (B); and decrease in relative uncertainty when sampling 100 more individuals for the observed catch structure (C) and uniform catch structure (D)**. Points in fig A and B represent the observed relative uncertainties.

The uncertainty curves (Fig 5A and 5B) for the three length classes for cod, and the effect on uncertainty when adding an extra 100 individuals to a given sample size (Fig 5C and 5D) show a clear relationship between the observed total sample sizes and the associated uncertainties. Regardless of the length class or catch structure, the uncertainties decreased with an increased sample size. Since the uncertainties follow a power-law, the slopes of the curves were much steeper for small sample sizes than for larger samples sizes. Furthermore, a relationship between the uncertainties and the strength of the data at a given length class was detected for the observed catch structure (Fig 5A). This relationship was not evident for the uniform catch structure (Fig 5B). The differences between the uncertainty curves were substantially reduced, showing a higher overlap of the observed uncertainties and their respective curves (Fig 5B). Moreover, the curves illustrating the effect of adding an extra 100 individuals showed similar patterns (Fig 5C and 5D). A large decrease in uncertainties was observed for sample sizes smaller than 250 individuals, while at sample sizes greater than 1000, the decrease was limited. As for the uncertainty curves, removing the effect of catch structure resulted in an overlap of the 3 different curves (Fig 5D), highlighting furthermore the importance of data strength at a given length class.

**Fig 6:**
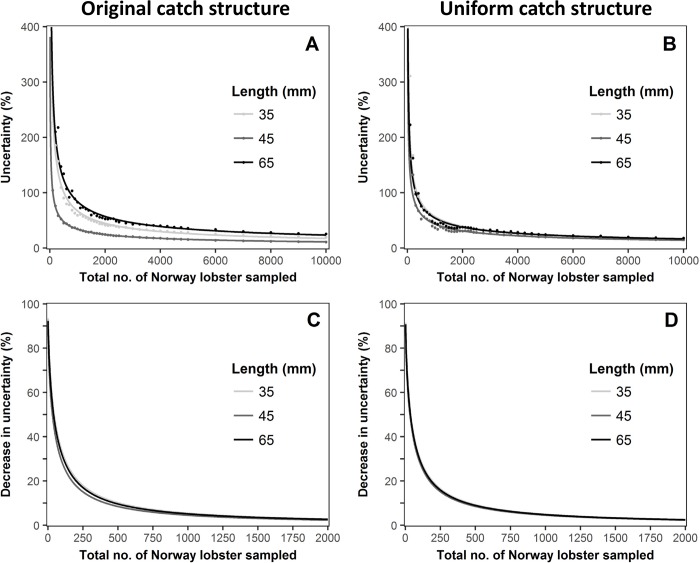
**Norway lobster uncertainty curves for the observed catch structure (A) and uniform catch structure (B); and decrease in relative uncertainty when sampling 100 more individuals for the observed catch structure (C) and uniform catch structure (D)**. Points in fig A and B represent the observed relative uncertainties.

The uncertainty curves for the three length classes for Norway lobster, and the effect on uncertainty when adding an extra 100 individuals to a given sample size are shown in Fig 6. Here, despite the catch ratio values at the given length classes being different for cod, similar results were observed. The uncertainties decreased with an increase in total sample size (Fig 6A and 6B); with the differences between the uncertainties curves being less pronounce for the uniform catch structure. The curves illustrating the effect of adding an extra 100 individuals showed very similar behaviour to those observed for cod (Fig 6C and 6D), where the overlap of the curves for the uniform catch structure was also pronounced (Fig 6D).

Using the length classes shown in Table 1, the average number of individuals required to obtain a given decrease in uncertainty by adding an additional 100 is shown in Table 2. A similar tendency for both species was observed; removing the effect of catch structure by using a uniform distribution reduced the average sample sizes obtained for each level of decrease in uncertainty, and the variability between length classes. Moreover, the values obtained from the original and uniform catch structures for both species are relatively similar, although Norway lobster showed a higher variability than cod. Based on the results and the methodology used to define the lower and upper bounds, a sampling effort of 500 to 1000 individuals is proposed for both species and catch structures.

**Table 2:** The number of individuals sampled and the associated decrease in uncertainty when measuring 100 more individuals. Mean values for all length classes considered and their respective standard deviations (in parenthesis) are presented.

	cod		Norway lobster	
Decrease in uncertainty	Original catch structure (*n*)	Uniform catch structure (*n*)	Original catch structure (*n*)	Uniform catch structure (*n*)
**50%**	51 (±11)	30 (±2)	49 (±29)	36 (±5)
**40%**	81 (±16)	51 (±3)	79 (±41)	60 (±7)
**30%**	133 (±23)	89 (±5)	129 (±60)	102 (±10)
**25%**	175 (±29)	119 (±6)	170 (±76)	137 (±13)
**20%**	238 (±37)	165 (±8)	231 (±98)	188 (±17)
**15%**	343 (±52)	243 (±11)	334 (±135)	274 (±23)
**10%**	555 (±80)	399 (±16)	540 (±209)	448 (±36)
**5%**	1190 (±164)	870 (±33)	1159 (±429)	971 (±73)
**2%**	3096 (±416)	2283 (±85)	3018 (±1090)	2539 (±186)
**1%**	6274 (±837)	4640 (±172)	6117 (±2191)	5154 (±374)

## Discussion

To achieve the objectives of the EU landing obligation, and those specified in the proposal for a new technical measures regulation (inter alia, flexibility and stakeholder involvement), greater flexibility in the number of gears able to be used in the fisheries is needed [2, 4]. A possible cost effective way to achieve these objectives is to have the industry involved in the development and testing of new gears, as well as the collection of data describing the selectivity of the new gears in relation to one which is legislated. However, to have a successful industry-collected data programme, those involved need to be burdened as little as possible as they have another objective, which is to carry out an economically viable fishery. Our results demonstrate that it is possible for fishermen to collect catch comparison data with a level of accuracy which makes it possible to correctly evaluate the performance of a new gear. With a relatively small sample size (500 to 1000 individuals per species) it is possible to assess the performance of a new fishing gear with an acceptable degree of uncertainty. These values are in the same range as those found by Herrmann *et al*. [18] for the estimation of selectivity curves using the paired-gear method; a method which is comparable to the catch comparison method. Considering that the total sample size per species is obtained throughout the testing phase, which usually has a duration of a couple of weeks, fishermen would need to sample relatively few individuals, per species, gear and haul, to obtain an acceptable degree of uncertainty. Furthermore, as the objective of a fishing gear selectivity trial is to address a specific issue in a fishery, the total number of sampled species is typically low, 2–3 species, including target and unwanted species. Therefore, on a haul level, the burden of collecting the required data should be minimal for the fishermen.

As a consequence of the low number of individuals required to be measured, the uncertainties around the average catch comparison curves are relatively large. The proposed number of individuals to measure is based on the analysis of the uncertainties associated with the different sampling efforts. As the results show, the uncertainties associated with the sampling effort follow a power law, whereby the differences between the overall uncertainties will be far greater for smaller sample sizes than for larger sample sizes. This behaviour of the uncertainties in relation to sample size was expected and confirmed by the overall quality of the power model fits (lowest *R*^*2*^ was 0.88) and was also described in Herrmann *et al*. [18]. Despite the proposed sampling range (500 to 1000 individuals) leading to potential large uncertainties, we consider it acceptable to use these for a preliminary assessment of the performance of a gear. However, more fish would need to be measured to reduce these uncertainties where the data are used for management purposes.

An additional aspect which needs to be considered is whether or not these results are applicable to other species and gear designs. Here we chose two species which are very different morphologically, and had very different observed population structures. The same trends in uncertainties were observed, e.g. large increases in uncertainties for the length classes with less data (tail area of the curves for the catch structures) for both species. To disclose the cause of this, uniform catch structures were simulated, which showed that the increase in uncertainties around the tails of the curves was caused by the low number of individuals measured at those length classes. Despite these differences, the uncertainty curves for both the observed and simulated catch structures were very similar to one another. Therefore, the results are applicable to different catch structures, and hence, species.

Despite the method being relevant to all species and areas, the difference in selectivity between the standard and test gears will define the magnitude of the effect and how early on in the sampling process an effect becomes evident. For example, in this study we looked at two different species, a target species (Norway lobster) where we did not expect to see an effect and a bycatch species (cod) where an effect was expected. For a species where no effect is expected, it should not matter if the entire catch is measured, as no effect should be present. However, for a species where a large effect is expected, one would anticipate the effect to be detectable early on in the sampling. This was the case observed in our study, where an effect for cod was detected after sampling only 100 individuals, while no effect was observed for Norway lobster at all sampling levels. This means that the magnitude of the effect is considerably more important than the species’ catch structure.

The importance of the magnitude means that if there is only a small effect of the new gear it might be difficult to detect a significant effect with so few individuals. However, when the objective is to define which gears should be taken to a full scientific trial, one would only want to choose those gears where a substantial effect can be obtained. Hence, this is not considered to be a major shortcoming of the method presented herein. Furthermore, a strength of this approach is that it is iterative, meaning that it is possible for the fishermen to collect additional data which can be analysed along the way. An additional strength of this approach is that if the modification which has been developed and tested does not achieve the desired outcome, additional modifications to the gear can be made without the need of an extensive gear selectivity trial.

The analysis presented herein is based solely on catch comparison data from a trawl fishery. Despite this, the methodology and findings may be applicable to different types of fisheries. The analysis presented here is based on length-dependent catch comparison data, where the performance of one gear is compared to a baseline. Therefore, the same approach should be applicable in different types of fisheries, for example, longline fisheries comparing different hook types or sizes, or creel fisheries with different mesh size. Hence, the findings of this paper should also be relevant to other types of fisheries.

In the present study, we do not consider between-haul variation, as an assumption of such variation may not be representative for other catch comparison experiments. Therefore, the range proposed here can be considered the absolute minimum number of individuals to measure, since one would expect the uncertainties to be larger when accounting for between-haul variation. However, since this is a first attempt at quantifying the minimum sampling efforts required to correctly document a gear’s relative selectivity, the values presented herein are still applicable as guidelines.

To be able to fully evaluate the results presented herein they should be benchmarked against those obtained from scientific trials. For example, are the trends observed in the industry-collected data the same as those observed in the scientific trial? This will help disclose whether or not these data can be used for a preliminary evaluation of a gears relative selectivity, and to what extent they can be used.

## Conclusion

This study shows that it is possible for fishermen to collect quality data on the performance of a new gear without affecting their main activity. This can help facilitate the needed flexibility in gear development which is currently lacking under the existing EU management system. By having the industry develop and test their own gears, as well as being involved in the collection of data, one increases the amount of potential solutions available to address the different issues emerging in the fisheries. From a list of potential solutions, the most promising and/or relevant ones can then be selected for a thorough scientific test in order to introduce the new gears into legislation. Here we have shown that it should be possible for the industry to collect relevant data to describe the relative selectivity of a new gear, where the guidelines presented herein can potentially be used in any fishery and for any gear. This new framework proposed here defines a new way of securing a fast and iterative development of thoroughly tested and well-documented fishing gears with minimal time and economic outlay.
